# Effects of stress relaxation in beta-titanium cantilevers used in orthodontic mechanics

**DOI:** 10.1590/2177-6709.26.6.e212069.oar

**Published:** 2021-12-15

**Authors:** Helder B. JACOB, Ariane S. GONZAGA, Brittany TRINH, Erik T. LE, Jeryl D. ENGLISH

**Affiliations:** 1 The University of Texas Health Science Center at Houston, School of Dentistry, Department of Orthodontics (Houston/TX, USA).; 2 Universidade Federal do Rio Grande do Norte, Departamento de Ortodontia (Natal/RN, Brazil).; 3 The University of Texas Health Science Center at Houston, School of Dentistry (Houston/TX, USA).

**Keywords:** Orthodontics, Corrective Orthodontics, Orthodontic wires

## Abstract

**Objective:**

This study evaluated the force decay and design shape changes caused by stress relaxation in two different orthodontic cantilever configurations.

**Methods:**

Eighty cantilevers made of 0.017 x 0.025-in beta-titanium wires were standardized in a passive position, using real scale templates, and randomly divided into two groups (n = 40): Type 1 and Type 2. Each group received a different design (Type 1 with three bends, and Type 2 with two bends), and both were divided in four subgroups (n = 10) according to the evaluation periods: G1 = 24h, G2 = 1 week, G3 = 4 weeks, and G4 = 8 weeks. Mechanical tests were performed immediately after preactivation and at the end of each period, to evaluate force decay. The cantilevers were also scanned and the angles of the bends were measured to assess shape changes.

**Results:**

Cantilever forces decayed over time. Type 1 - G1 showed less force decay than Type 2 (10.83 cN vs 17.87 cN). Type 1 cantilevers showed significant force decay only when G4 was compared to G1 (9.05 cN), G2 (11.73 cN), and G3 (9.78 cN). Type 2 cantilevers presented differences when G1 was compared to G2 (9.57 cN) and G3 (7.89 cN). Regarding to the cantilever angle closest to the bracket insertion, Type 1 cantilevers showed significant decrease for G2 (1.58°) and G4 (1.52°).

**Conclusions:**

Cantilevers’ design and proximity of the bends influenced force decay pattern overtime. Type 1 cantilevers presented more stable design at the first weeks than Type 2.

## RESUMO

**Objetivo:**

O presente estudo avaliou a perda de força e as mudanças no *design* causadas pelo relaxamento da tensão em duas diferentes configurações de *cantilevers* ortodônticos.

**Métodos:**

80 *cantilevers* feitos de fios de beta-titânio 0,017” x 0,025” foram padronizados em uma posição passiva, usando modelos em escala real, e divididos aleatoriamente em dois grupos (n = 40) com desenhos diferentes: o Tipo 1 possuía três dobras e o Tipo 2, duas dobras. Ambos os tipos foram divididos em quatro subgrupos (n = 10), de acordo com os períodos de avaliação (G1 = 24h; G2 = uma semana; G3 = quatro semanas; G4 = oito semanas). Testes mecânicos foram feitos imediatamente após a pré-ativação e ao fim de cada período, para avaliar a perda de força. Os *cantilevers* também foram escaneados e os ângulos das dobras foram medidos, para se avaliar as mudanças em seu formato.

**Resultados:**

Os *cantilevers* perderam força ao longo do tempo. Os do Tipo 1 - G1 mostraram menos perda de força do que os do Tipo 2 (10,83 cN *vs* 17,87 cN). Os*cantilevers* do Tipo 1 mostraram perda de força significativa apenas ao se comparar G4 a G1 (9,05 cN), G2 (11,73 cN) e G3 (9,78 cN). Os *cantilevers* do Tipo 2 apresentaram diferenças quando G1 foi comparado a G2 (9,57 cN) e G3 (7,89 cN). Com relação ao ângulo do cantiléver mais próximo à inserção no braquete, os *cantilevers* do tipo 1 mostraram redução significativa para G2 (1,58°) e G4 (1,52°).

**Conclusões:**

O *design* dos *cantilevers* e a proximidade das dobras influenciaram o padrão de perda de força ao longo do tempo. Os *cantilevers* do Tipo 1 mostraram um *design* mais estável nas primeiras semanas do que os do Tipo 2.

## INTRODUCTION

Orthodontic tooth movement is the result of force application on a tooth. Primarily, orthodontists generate forces using archwires, springs, and elastics. The role of the orthodontic wire is to act as a spring and/or a guide, and cantilevers appear as a versatile tool for orthodontics.

Cantilevers can be a simple straight wire or a wire with a special shape. Anchored at only one end, a cantilever is a beam with which the orthodontist can easily and accurately predict tooth movement.^1^ ^-^ ^3^ By producing effects on the tooth in all three planes, controlling and individualizing the forces applied, cantilevers can be applied to provide intrusion or extrusion of one or several teeth simultaneously. They can also perform tractions, uprightings, retractions, and early corrections of the deep curve of Spee.^1^ ^-^ ^6^ Because of their formability and springback characteristics, titanium-molybdenum alloys, also called β-titanium, are often used for the manufacturing of cantilevers.^1^ ^-^ ^5^ ^,^ ^7^ ^-^ ^10^ This alloy in straight-wire applications can be deflected 105 percent more than stainless steel without permanent deformation, and its stiffness makes it ideal in applications where less force is required but a lower modulus of elasticity would be inadequate to develop the required force magnitudes.^11^ Therefore, the use of β-titanium alloys enables the construction of cantilevers with simpler designs, saving time during the clinical procedures. 

Despite their wide use, the best interval between activation is difficult to determine. The choice of the design of the segmented arch, material, and the way that it is bent has direct influence on how forces decay through time during clinical use.^7^ ^-^ ^10^

The shape of the cantilever as well as its activation are individual choices of each orthodontist, and often the simpler shapes are more frequently used. However, the impact of a cantilever’s design on the delivery of forces is a variable that is more frequently studied in more complex designs, and there is a lack of evidence for those with simpler designs, as well as which is the best form of use during orthodontic treatment. Therefore, the present study aims to compare the force decay and design shape changes caused by stress relaxation, as well as to determine the ideal time interval of reactivation, between two different cantilevers’ activation types.

## MATERIAL AND METHODS

**Figure 1:**
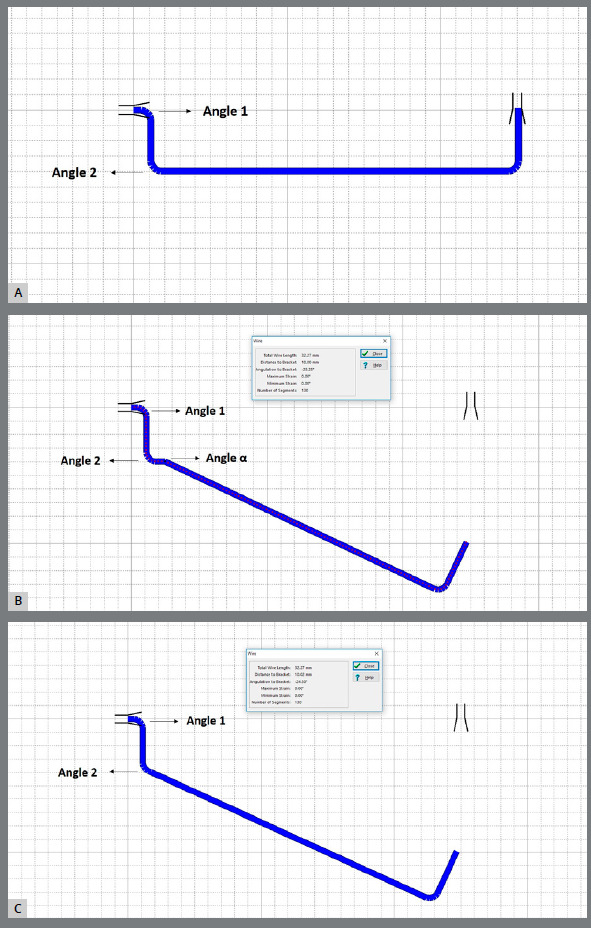
A) Template developed in the Loop software (dHAL Orthodontic Software) for Type 1 and Type 2 cantilevers. B) Preactivation template for the Type 1 cantilever. C) Preactivation template for the Type 2 cantilever.

Eighty cantilevers, made of 0.017 x 0.025-in beta-titanium wires (American Orthodontics, Sheboygan, WI), were hand-bent by one calibrated operator using a Marcotte plier (Hu-Friedy, Chicago, IL). The cantilevers, with dimensions of 5 mm in height and 25 mm in length, were standardized in a passive position using real scale templates (Fig 1) generated by the Loop software v. 1.7.0.0 (Orthodontic Loop Simulator- Hellas, Greece).^12^

**Figure 2:**
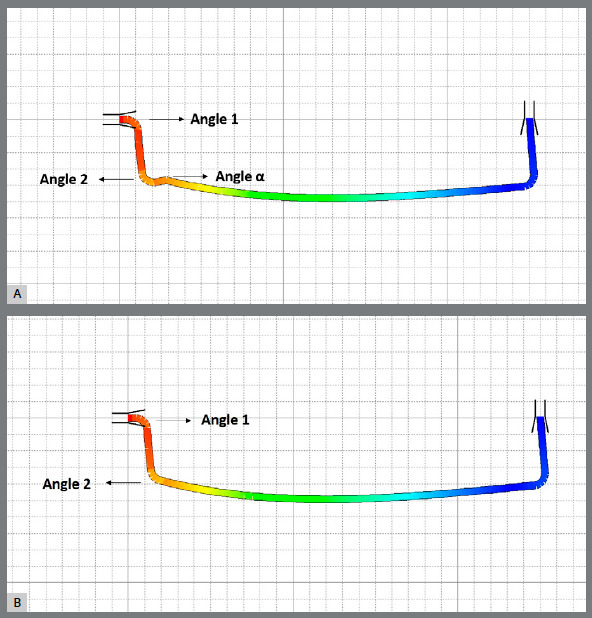
Loop software simulation of tension concentration areas for: A) Type 1 cantilever and B) Type 2 cantilever. The gradient of colors change from red, which means maximum tension concentration, to blue, which represents the minimum tension concentration.

Afterward, the cantilevers were randomly divided into two groups (n = 40) and submitted to two different preactivations designs: Type 1, with an extra bend for activation (angle α); and Type 2 (Fig 2). 

Then, using a template generated by the Loop software in a 1:1 scale for standardization, the Type 1 cantilevers and the Type 2 cantilevers were preactivated 10 mm using the Marcotte plier. The amount of activation was chosen to provide enough moment for molar uprighting due to the cantilever length;^13^ in addition, the amount of activation could be used for intrusion of the mandibular anterior teeth.^14^ ^,^ ^15^ According to the software, Type 1 cantilevers should release 46.25 centinewtons (cN) and Type 2 cantilevers should release 44.41 cN. Each group type was divided in four subgroups (n = 10), according to their evaluation periods after the preactivation: group 1 (G1) = one day, group 2 (G2) = one week, group 3 (G3) = four weeks, and group 4 (G4) = eight weeks.

Immediately after the cantilevers were preactivated (baseline), they were tested using a tabletop universal testing machine (Series 4400 System, Instron, Norwood, MA), equipped with a load cell of 10^3^cN (load accuracy of 0.5%), at the testing speed of 5.0 mm per minute. Bluehill 2 Universal static testing software was used to set variables of the test and export data. Right after the mechanical test, the cantilevers were scanned with an HP scanner (Scanjet 3670, Hewlett-Packard, Palo Alto, CA) at high resolution (600 dpi), and each one of the angles was measured by the same operator, using Iconico Screen Protractor (Iconico, New York, USA).

**Figure 3:**
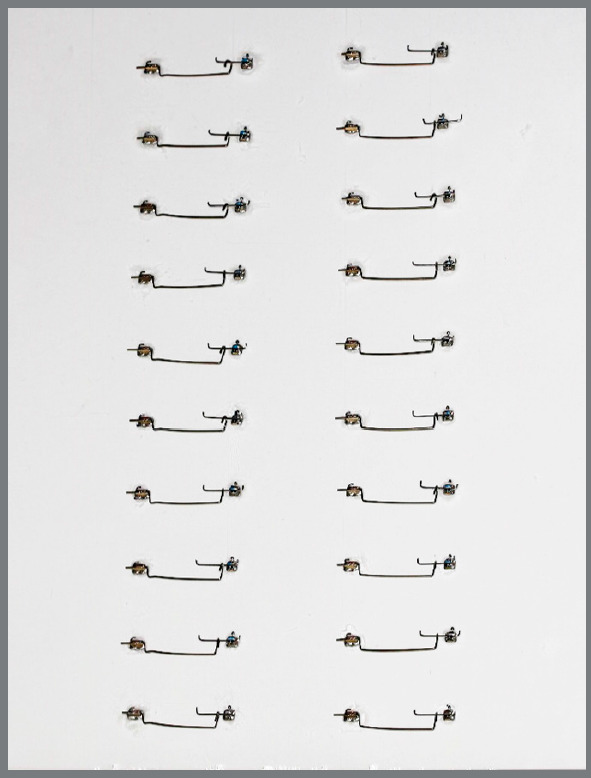
Custom made device, simulating the clinical activation of cantilevers, in order to keep the cantilevers activated.

**Figure 4:**
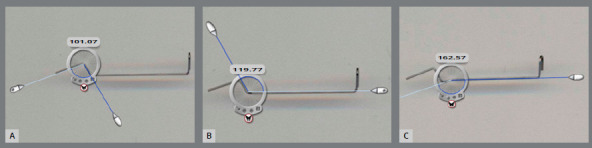
Measurement of the angles: A) angle 1; B) angle 2, and C) angle α.

After being scanned, the cantilevers were maintained active (simulating the clinical activation) in a custom-made device (Fig 3). The device was composed of a plastic sign-holder with brackets and tubes (0.018-in slot) from the SPEED System (Strite Industries, Ontario, Canada) carefully bonded with Loctite Super Glue (Loctite, Westlake, OH) at a distance of 28 mm. A 10 mm 0.016 x 0.022-in stainless steel wire was inserted on the brackets, and the cantilevers were hooked onto it. The bonding was organized in two rows of ten pairs, separated by design type at each side of the sign-holder, and kept ordered throughout the experiment. The cantilevers were prepared, stored, and tested at room temperature (set at 23°C). After performing mechanical test for each group, the cantilevers were scanned and the angles were measured by the same operator, in order to assess the permanent deformation of the structure (Fig 4).

### STATISTICAL ANALYSIS

Each measurement was taken twice at one-week intervals, for error assessment. Intraobserver random error was estimated using intraclass correlation coefficient (ICC) and method errors [√(∑d^2^/2n)], and systematic differences were accessed using a paired *t*-test. The average of the first and second measurements was used for statistical analysis. To detect stress relaxation of the angles, force decay, differences between designs, and differences between groups, *t*-test, paired *t*-test, ANOVA one-way, and *post-hoc* Tukey’s test were used. All statistical procedures were performed using IBM SPSS™ software (version 25.0, SPSS, Armonk, NY), with a significance level of 0.05.

## RESULTS

### FORCE ANALYSIS

Intraobserver systematic errors of the two measurement moments did not show significant differences. The differences between the first and second measurement ranged from <0.01 cN to 1.16 cN. Method errors ranged from 0.69 cN to 1.80 cN. Interclass correlations (ICC) from 0.694 to 0.992 were consistently high.

The intragroup and intergroup analysis of forces showed no significant differences between the types in all four groups at the baseline (Table 1). Force delivered initially by the Type 1 cantilevers ranged from 35.43 cN to 41.00 cN, whereas the Type 2 cantilevers delivered initial forces ranging from 35.78 cN to 42.20 cN.

**Table 1:** Descriptive statistics and comparison of forces at activation (baseline).

	Type 1		Type 2		Type 1 x Type 2
	Mean	S.D.	Mean	S.D.	Prob.
Group 1	37.28	6.67	42.20	4.98	0.078
Group 2	36.15	7.53	37.84	3.60	0.529
Group 3	35.43	4.02	39.02	6.68	0.164
Group 4	41.00	6.14	35.78	6.99	0.093

**Table 2:** Descriptive statistics and intergroup comparison of changes of forces through time.

	Type 1			Type 2			Type 1 x Type 2
	Changes	S.D.	Prob.	Changes	S.D.	Prob.	Prob.
Group 1	10.83	5.03	*<0.001*	17.87	4.72	*<0.001*	*0.005*
Group 2	8.15	3.87	*<0.001*	8.29	3.01	*<0.001*	0.926
Group 3	10.09	2.57	*<0.001*	9.98	6.26	*0.001*	0.957
Group 4	19.88	5.81	*<0.001*	12.47	9.76	*0.003*	0.054

Bold and italic indicate statistically significant differences between replicates (p < 0.05). Obs.: Changes were calculated by initial force minus the final force within each group.

Although there was a significant decrease of force among all groups in both Type 1 cantilevers and Type 2 cantilevers, only the 1-day period evaluation showed significant difference between the two cantilevers design (Table 2). After the 1-day period, the vertical force decreased 10.83 cN and 17.87 cN for Type 1 and Type 2 cantilevers, respectively.

Over time, Type 1 cantilevers showed significant force decay among three out of six comparisons (Table 3); the force decayed significantly more for G4 (8-week period) when compared to G1 (9.05 cN), G2 (11.73 cN), and G3 (9.78 cN). Type 2 cantilevers showed significant force decay only for two evaluations among groups (Table 3); the vertical force decayed more for G1 in comparison to G2 (9.57 cN) and G3 (7.89 cN).

**Table 3:** Intragroup comparison of changes of forces through time.

Changes		Type 1			Type 2		
		Mean diff.	S.E.	Prob.	Mean diff.	S.E.	Prob.
Group 1	Group 2	2.68	1.97	0.532	9.57	2.37	*0.001*
	Group 3	0.73	1.97	0.982	7.89	2.37	*0.011*
	Group 4	-9.05	2.00	*< 0.001*	5.40	2.88	0.256
Group 2	Group 3	-1.95	2.00	0.768	-1.68	2.88	0.936
	Group 4	-11.73	2.00	*< 0.001*	-4.18	2.88	0.477
Group 3	Group 4	-9.78	2.00	*< 0.001*	-2.49	2.88	0.822

Bold and italic indicate statistically significant differences between replicates (p < 0.05). Obs.: Mean difference (Mean diff.) was calculated based on first column group minus second column group, i.e., Mean diff. = Group 1 - Group 2.

### ANGLE ANALYSIS

Intraobserver systematic errors showed statistically significant differences (*p*< 0.05) in 7 out of 28 measurements. Type 1 cantilevers in the G1 presented 43% of the systematic errors, and 1.4º was the largest systematic error. Method errors ranged from 0.402º to 1.520º. Interclass correlations (ICC) ranged from 0.434 to 0.966.

Type 1 cantilevers showed significant changes in 4 out of 12 angles during the experiment (Table 4). Angle 1 showed significant opening in G2 (-1.58°) and in G4 (-1.52°). Angle 2 had significant closure in G3 (0.82°), and angle α showed significant opening in G2 (-0.85°).

**Table 4:** Changes of the angles of Type 1 cantilevers through time.

Type 1	Angle 1					Angle 2					Angle α				
	Initial		Changes		Prob.	Initial		Changes		Prob.	Initial		Changes		Prob.
	Mean	S.D.	Diff.	S.D.		Mean	S.D.	Diff.	S.D.		Mean	S.D.	Diff.	S.D.	
Group 1	98.43	2.48	-0.53	0.92	0.098	97.65	1.82	0.20	1.40	0.670	160.71	1.98	-0.47	1.35	0.298
Group 2	96.68	2.30	-1.58	0.72	*<0.001*	96.89	1.35	-0.93	1.51	0.083	160.33	2.09	-0.85	0.85	*0.011*
Group 3	96.67	2.30	-0.05	0.75	0.824	96.91	1.65	0.82	0.073	*0.006*	159.65	2.15	-0.70	1.01	0.054
Group 4	96.46	2.25	-1.52	0.82	*<0.001*	97.01	1.75	0.84	1.65	0.141	159.50	2.82	-0.51	1.16	0.195

Bold and italic indicate statistically significant differences (p < 0.05). Obs.: Changes were calculated by initial force minus the final force within each group.

Regarding Type 2 cantilevers, Angles 1 and 2 changed significantly through time (Table 5). Angle 1 showed a progressive opening, with significant differences at G1 (-0.74°), G2 (-0.88°) and G4 (-1.42°); while Angle 2 showed a progressive closing, with significant differences at G2 (0.70°), G3 (0.69°) and G4 (0.97°).

**Table 5:** Changes of the angles of Type 2 cantilevers through time.

Type 2	Angle 1					Angle 2				
	Initial		Changes		Prob.	Initial		Changes		Prob.
	Mean	S.D.	Diff.	S.D.		Mean	S.D.	Diff.	S.D.	
Group 1	98.00	3.42	-0.74	0.94	*0.034*	116.68	2.52	0.73	1.19	0.083
Group 2	95.69	1.52	-0.88	0.90	*0.013*	116.78	1.49	0.70	0.71	*0.012*
Group 3	95.80	2.71	-0.49	0.85	0.100	115.87	2.54	0.69	0.51	*0.002*
Group 4	96.00	1.45	-1.42	0.66	*< 0.001*	117.57	2.04	0.97	0.55	*< 0.001*

Bold and italic indicate statistically significant differences (p < 0.05). Obs.: Changes were calculated by initial force minus the final force within each group.

Comparing groups, Type 1 angle’s changes were different through time for Angles 1 and 2 (Table 6), while Type 2 cantilevers did not present differences between groups for either angle (Table 7). Angle 1 of the Type 1 cantilever showed significant differences among four out of six comparisons. The changes were significant for G2 in comparison to G1 (1.04°) and G3 (-1.52°), and for G3 x G4 (1.47°), while the difference between changes at G2 and G4 was minimal (-0.06°). Angle 2 showed that changes after 1 week (G2) were significant when compared to G3 (-1.75°) and G4 (-1.77°), and angle α did not express changes among groups.

**Table 6:** Intragroup comparison of the change of the angles of Type 1 cantilevers.

		Angle 1			Angle 2			Angle α		
		Mean diff.	S.E.	Prob.	Mean diff.	S.E.	Prob.	Mean diff.	S.E.	Prob.
Group 1	Group 2	1.04	0.36	0.031	1.12	0.61	0.274	0.38	0.50	0.866
	Group 3	-0.48	0.36	0.551	-0.62	0.61	0.738	0.23	0.50	0.965
	Group 4	0.99	0.36	0.046	-0.65	0.61	0.718	0.04	0.50	0.999
Group 2	Group 3	-1.52	0.36	0.001	-1.75	0.61	0.034	-0.15	0.50	0.990
	Group 4	-0.06	0.36	0.998	-1.77	0.61	0.031	-0.34	0.50	0.903
Group 3	Group 4	1.47	0.36	0.001	-0.02	0.61	0.999	-0.19	0.50	0.980

Bold and italic indicate statistically significant differences (*p* < 0.05). Obs.: Mean difference (Mean diff.) was calculated based on first column group minus second column group, i.e., Mean diff. = Group 1 - Group 2.

**Table 7:** Intragroup comparison of the change of the angles of Type 2 cantilevers.

		Angle 1			Angle 2		
		Mean diff.	S.E.	Prob.	Mean diff.	S.E.	Prob.
Group 1	Group 2	0.14	0.38	0.983	0.03	0.35	0.999
	Group 3	-0.25	0.38	0.908	0.04	0.35	0.999
	Group 4	0.68	0.38	0.289	-0.24	0.35	0.903
Group 2	Group 3	-0.39	0.38	0.729	0.01	0.35	0.999
	Group 4	0.54	0.38	0.487	-0.27	0.35	0.867
Group 3	Group 4	0.93	0.38	0.082	-0.28	0.35	0.856

Bold and italic indicate statistically significant differences (*p* < 0.05). Obs.: Mean difference (Mean diff.) was calculated based on first column group minus second column group, i.e., Mean diff. = Group 1 - Group 2.

## DISCUSSION

Cantilever forces decayed over time. Independently of the design, both cantilevers presented approximately a 20 cN decrease in force over the 8-week period. Maximum force decay was approximately 48% and 42% for Type 1 cantilevers and Type 2 cantilevers, respectively. Studies related to orthodontic springs and archwires have shown significant force decay over observational periods between the measurements made at the baseline and eight weeks of experiment, with maximum force decay between 26% and 29% for more complex configuration such as “T” loops.^7^ ^-^ ^10^ This interaction of time on the decreased rate of force of the loops also means that the load-deflection rate of the loops decreased along the experimental period, although force changes did not obey a linear decrease.^7^ ^-^ ^10^

The cantilever’s design influenced force decay pattern over time. Although the maximum force decay difference between cantilevers was only 2 cN, the Type 1 cantilevers presented the maximum force decay after a long period of time (8 weeks) while the Type 2 cantilevers presented their maximum force decay after a short period of time (24 hours). The cantilevers with the extra bend (Type 1) presented less force decay (61%) within 24 hours than Type 2 cantilevers, and more force decay after 8-week period (60%) than the cantilever with a traditional design (Type 2 cantilever). These findings agree with several other reports that described the effect of force decrease over time on straight wires and in more elaborate configurations.^7^ ^,^ ^9^ ^,^ ^16^ ^,^ ^17^ The force decay over time for both types of cantilevers can be explained by the stress relaxation phenomenon.

Stress relaxation can be defined as the deformation as a function of time. This time-related deformation, also called *creep,* is the result of an increase in strain or a decrease in stress caused by microscopic progressive movement of dislocations in the crystalline structure of high stressed metals.^7^ ^,^ ^18^ ^-^ ^20^ In Orthodontics, bends are placed in archwires to facilitate particular tooth movements. However, these bends concentrate stress that lead to *creep.* ^7^ Previous studies looked upon this effect in more elaborate configurations, evaluating stress relaxation on T-Loops springs preactivated by bends, and revealing a decrease on the force levels over time according to the bends’ shapes.^7^ ^-^ ^10^

Sharpness and proximity of bends influence force decay. Type 1 cantilever design presents three concentrated bends in one end, and Type 2 cantilever design presents only two, and the changes of forces could be explained by the differences between the cantilevers’ designs and its tension concentration areas (Fig 2). The different designs led to more force decay within eight weeks, and earlier changes of the angles of Type 1 than Type 2. Type 1 showed significant changes of the angles from one period of time evaluation to another, with abrupt opening of the Angle 1 at one week (G2) and at the end of experiment (G4); and Type 2 showed significant progressive opening of Angle 1 and closing of Angle 2. These angle changes show the over time deactivation of the cantilevers, noticed as the decrease of forces. Beyond the force decay of the preactivated loops, some studies^7^ ^,^ ^9^ ^,^ ^10^ stated that the sharpness of the bend influences the final angles of the spring’s structure. In addition, concentrated bends are also responsible for relaxation and/or plastic deformation over time. Structural areas of the springs can be affected by the proximity of the bends and/or stress-relaxation, as previously reported by the literature using “T” loop shape designs.^7^ ^,^ ^9^ ^,^ ^10^

Normally, the orthodontist schedules the patient’s appointments from four to eight weeks apart, and based on this study, the Type 1 cantilevers showed a more stable design than Type 2, due to amount of force decreasing in the first few weeks. The effect of stress relaxation was gradual in Type 1 cantilevers, occurring majorly after 4 weeks. The deformation presented during the first 24 hours in the Type 1 cantilever decreased the force level in approximately 29%, whereas the Type 2 cantilever decreased the force level in approximately 42%. Then, the reactivation could be performed after 4 weeks to maintain these optimal forces producing a more constant force levels. Although these results suggest that reactivation of cantilevers could be done up to 8 weeks, it is important to consider the permanent deformation that beta-titanium wires suffer as a function of time when exposed to long periods of deflection.^7^ ^,^ ^9^ ^,^ ^10^ ^,^ ^16^ ^,^ ^17^ ^,^ ^21^

Clinically, a force decay of 20 cN has a great influence over tooth movement. Light forces are used for intrusion and it is recommended approximately 60cN to intrude all mandibular incisors.^14^ If the force decay to 25 cN (20cN less than the applied initially), the intrusion of the mandibular incisors would not be performed. Another clinical situation is related to the moment generate by the applied force. In order to efficiently upright one molar, it is necessary to produce a moment with magnitude of approximately 1100 cN-mm.^13^ Considering a cantilever length of 25 mm, a vertical force of approximately 45 cN is required at the point of force application. A decay of 20 cN over the initial 45 cN will not provide enough force to generate the ideal moment.

The results of this study need to be cautiously interpreted. Performing further tests to confirm this information, such as the measurement of forces over time after reactivation of the same cantilevers and clinical research (*in vivo*) of stress relaxation of cantilevers during the orthodontic treatment, is necessary to corroborate the findings of the present study. In addition, to better understand the cantilevers’ behavior, X-ray diffraction test should be used to analyze the changes on the surface characteristics of the crystallographic structure. 

## CONCLUSIONS

Within the limits of this study, the following conclusions can be drawn:

» Cantilever’s design influences force decay pattern over time.
» Sharpness and proximity of the bends influences force decay.
» Type 1 cantilevers have a more stable design than Type 2, related to force level at first few weeks.
» Type 1 cantilevers should be reactivated after 4 weeks.

## References

[1] Caballero GM, Carvalho OA, Hargreaves BO, Brito HH, Magalhães PA, Oliveira DD (2015). Mandibular canine intrusion with the segmented arch technique A finite element method study. Am J Orthod Dentofacial Orthop.

[2] Raveli TB, Raveli DB, de Mathias Almeida KC, Pinto ADS (2017). Molar uprighting a considerable and safe decision to avoid prosthetic treatment. Open Dent J.

[3] Lima APB, Costa PA, Barbosa NMV, Alemida-Pedrin RR, Paranhos LR, Cardoso M de A (2019). Segmented mechanics for traction of impacted maxillary canine case report with a 3-year follow-up. Biosci J.

[4] Majourau A, Norton LA (1995). Uprighting impacted second molars with segmented springs. Am J Orthod Dentofacial Orthop.

[5] Sawicka M, Racka-Pilszak B, Rosnowska-Mazurkiewicz A (2007). Uprighting partially impacted permanent second molars. Angle Orthod.

[6] Martins RP (2017). Early vertical correction of the deep curve of Spee. Dental Press J Orthod.

[7] Caldas SG, Martins RP, Viecilli RF, Galvão MR, Martins LP (2011). Effects of stress relaxation in beta-titanium orthodontic loops. Am J Orthod Dentofacial Orthop.

[8] S R, Caldas SG, Martins LP, Martins RP (2016). Effects of stress relaxation in beta-titanium orthodontic loops Part II. Angle Orthod.

[9] Caldas SGFR, Martins RP, Araújo ME, Galvão MR, Silva RSD, Martins LP (2017). Stability of beta-titanium T-loop springs preactivated by gradual curvature. Dental Press J Orthod.

[10] Viecilli AF, Freitas MPM (2018). The T-loop in details. Dental Press J Orthod.

[11] Burstone CJ, Goldberg AJ (1980). Beta titanium a new orthodontic alloy. Am J Orthod.

[12] Halazonetis DJ (1997). Design and test orthodontic loops using your computer. Am J Orthod Dentofacial Orthop.

[13] Romeo DA, Burstone CJ (1977). Tip-back mechanics. Am J Orthod.

[14] van Steenbergen E, Burstone CJ, Prahl-Andersen B, Aartman IH (2005). The influence of force magnitude on intrusion of the maxillary segment. Angle Orthod.

[15] Ren Y, Maltha JC, Kuijpers-Jagtman AM (2003). Optimum force magnitude for orthodontic tooth movement a systematic literature review. Angle Orthod.

[16] Hudgins JJ, Bagby MD, Erickson LC (1990). The effect of long-term deflection on permanent deformation of nickel-titanium archwires. Angle Orthod.

[17] Hanyuda A, Nagasaka S, Yoshida T (2006). Long-term time effect on load-deflection characteristics of orthodontic wires. Orthod Waves.

[18] Earthman J (2000). ASM Handook: mechanical testing and evaluation.

[19] Anusavice KJ, Brantley WA, Anusavice KJ (2003). Phillips science of dental materials.

[20] Callister WD, Rethwisch DG (2014). Materials science and engineering: an introduction.

[21] Wong EK, Borland DW, West VC (1994). Deformation of orthodontic archwires over time. Aust Orthod J.

